# Characterization of NKG2-A/-C, Kir and CD57 on NK Cells Stimulated with pp65 and IE-1 Antigens in Patients Awaiting Lung Transplant

**DOI:** 10.3390/life12071081

**Published:** 2022-07-19

**Authors:** Laura Bergantini, Miriana d’Alessandro, Ambra Otranto, Dalila Cavallaro, Sara Gangi, Antonella Fossi, Felice Perillo, Luca Luzzi, Edoardo Zanfrini, Piero Paladini, Piersante Sestini, Paola Rottoli, Elena Bargagli, David Bennett

**Affiliations:** 1 Respiratory Disease Unit, Department of Medical Sciences, University Hospital of Siena, Azienda Ospedaliera Universitaria Senese (AOUS), Viale Bracci, 53100 Siena, Italy; laurabergantini@gmail.com (L.B.); ambra.otranto@gmail.com (A.O.); cavallarodalila@gmail.com (D.C.); sara.gangi@student.unisi.it (S.G.); antonella.fossi@gmail.com (A.F.); felice.perillo87@gmail.com (F.P.); sestini@unisi.it (P.S.); paola.rottoli@unisi.it (P.R.); bargagli2@gmail.com (E.B.); david.btt@gmail.com (D.B.); 2 Respiratory Disease and Lung Transplant Unit, Department of Medical Sciences, Surgery and Neurosciences, Siena University, 53100 Siena, Italy; 3 Thoracic Surgery Unit, Cardio-Thoracic and Vascular Department, University Hospital of Siena, Azienda Ospedaliera Universitaria Senese (AOUS), 53100 Siena, Italy; dr.luca.luzzi@gmail.com (L.L.); edoardo.zanfrini@gmail.com (E.Z.); piero.paladini@unisi.it (P.P.)

**Keywords:** NK cells, T cells, immunology, lung transplant, cytomegalovirus

## Abstract

Introduction: Cytomegalovirus (CMV) is the leading opportunistic infection in lung transplant (LTx) recipients. CMV is associated with graft failure and decreased survival. Recently, new antiviral therapies have been proposed. The present study aimed to investigate NK and T cell subsets of patients awaiting LTx. We analyzed the cellular populations between reactive and non-reactive QuantiFERON (QF) CMV patients for the prediction of immunological response to infection. Methods: Seventeen pre-LTx patients and 15 healthy controls (HC) have been enrolled. QF and IFN-γ ELISA assay detections were applied. NK cell subsets and T cell and proliferation assay were detected before and after stimulation with pp-65 and IE-1 CMV antigens after stratification as QF+ and QF−. Furthermore, we quantified the serum concentrations of NK− and T-related cytokines by bead-based multiplex analysis. Results: CD56^br^CD16^low^NKG2A^+^KIR^+^ resulted in the best discriminatory cellular subsets between pre-LTx and HC. Discrepancies emerged between serology and QF assay. Better proliferative capability emerged from patients who were QF+, in particular in CD8 and CD25-activated cells. CD56^br^CD16^low^, adaptive/memory-like NK and CD8Teff were highly increased only in QF+ patients. Conclusions: QF more than serology is useful in the detection of patients able to respond to viral infection. This study provides new insights in terms of immunological responses to CMV in pre-LTX patients, particularly in NK and T cells biology.

## 1. Introduction

Lung transplant (LTX) is considered the best therapeutic option for a variety of end-stage and progressive lung diseases [1]. Cytomegalovirus (CMV) is the leading opportunistic infection in solid organ transplant, particularly in lung transplant patients. It is associated with graft failure and shorter overall survival [2].

In recent years, CMV management in LTX patients has become a challenge for research. New findings include better understanding of diagnostic thresholds, optimized methods of prevention, and new antiviral therapies, leading to the better management of CMV infection before and after transplant [3,4,5,6]. However, certain molecular and immunological aspects are still unclear. CMV affects the host immune system at different levels and has simultaneous pro-inflammatory and anti-inflammatory effects [7]. Different cellular subsets play a key role in response to viral infection, first of all Natural Killer (NK) cells.

NK cells are important cytotoxic innate lymphocytes that contribute to infection control, malignancy and autoimmunity. They express a variety of inhibitory and activating cell-surface receptors [8]. Human NK cells are usually divided into two major populations based on the expression of CD56 and CD16: CD56^bright^CD16^dim^ and CD56^dim^CD16^bright^. The CD56^bright^CD16^dim^ subset that expresses NKG2A and is devoid of killer cell immunoglobulin-like receptors (KIRs) is considered the most immature phenotype, while the mature CD56^dim^CD16^bright^ subset is associated with different proportions of KIRs, NKG2A and NKG2C [9,10]. In particular, CD56^dim^CD16^bright^ NK cells can be roughly divided into maturing (NKG2AposKIRneg), double positive (NKG2A^pos^KIR^pos^), and mature (NKG2A^neg^KIR^pos^LILRB1^pos^) NK cells. In the latter subpopulation, the most mature KIRposCD16bright NK cells also express CD57 and are considered to be the terminally differentiated compartment [11]. Researchers also demonstrated that the role of NKG2C and CD57 selectively expanded following exposure to CMV expression on the NK subset. For these reasons CD57+NKG2C+ NK frequency serves to measure NK responses against HCMV [12,13].

Recently, it was found that after the first contact with CMV, a specific subtype of NK is generated followed by long-lived “memory” NK cells that are more protective during a second encounter with the pathogen: this subset expresses CD57, which is clonally expanded by infections [14].

The NK cell response to human CMV has provided important insights into how NK cells specifically recognize and control viral infection and how infection affects NK cells [15].

However, it is also important to consider T cell compartments, which play a key role in defense against extrinsic (e.g., pathogens) and intrinsic factors (e.g., malignant cells) [16]. T effector (Teff) (CD45RA^−^) and T central memory (Tcm) (CD45RA^+^) subsets expressing CD62L^−^CD27^+/−^ and CD62L^++^CD27^++^, respectively, have unique characteristics that give them differential migratory capacity, longevity and function [17].

They develop from the naive (Tn) (CD27^+++^CD62L^+++^) repertoire by activation [18,19], which is based on the expression of different surface markers, cytokine release and transcription factors.

Due to the unknown link between NK and adaptive immunity against HCMV, our primary aim was to analyze the different cell phenotypes and functions, including NK and T cell subsets, of patients awaiting lung transplant. Our second aim was to analyze and compare cell populations in patients reactive and non-reactive to CMV with a view to predicting their immunological response to CMV infection.

## 2. Materials and Methods

### 2.1. Patients and Study Design

Seventeen consecutive LTX patients placed on the lung transplant waiting list at the Siena Regional Referral Centre for Lung Transplant between March 2020 and July 2021 were enrolled in the study, together with 15 healthy controls. All medical and personal data were entered in an electronic database.

**Figure 1:**
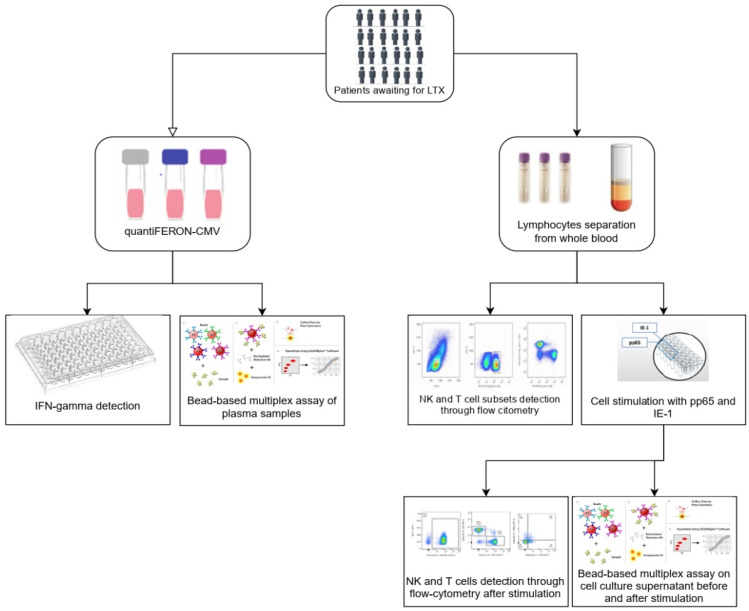
Study design.

The eligibility criteria for the waiting listing were evaluated in patients by two expert clinicians according to international guidelines [20]. The exclusion criteria were family history, underlying inflammatory diseases, and active CMV infection or associated disease at the time of sapling. All included patients had detectable pre-transplant anti-CMV IgG titers, resulting seropositive. The flow chart of the study is reported in Figure 1.

All patients gave their written informed consent to participate in the study, which was approved by our local ethics committee (CEAVSE, *Markerlung 17431*, Respir1, Prot no. 15732, 16 September 2019).

### 2.2. QuantiFERON-CMV Assay and INF-γ Detection

The QuantiFERON-CMV (QF) assay was performed according to the manufacturer’s instructions (Cellestis, a QIAGEN company, Melbourne, Australia). All patients had HLA class I alleles capable of binding CMV peptides. Supernatants were analyzed for IFN-γ (IU/mL) by ELISA. A CMV antigen result is “Positive” when the CMV antigen response minus the negative control response is >0.2 IU/mL IFNγ, and it is defined as QF+. According to the manufacturer, the result is “Indeterminate” when the same parameter is <0.2 IU/mL (it is defined as QF+/−) and the IFNγ level in the mitogen tube minus that of the negative control is <0.5 IU/Ml, and it is defined as QF−.

### 2.3. Preparation and Storage of PBMCs

Cytofluorimetric analysis was performed at the Siena University Respiratory Diseases Laboratory in the period January 2021–June 2021. Lymphocyte subset percentages in PBMCs from patients and controls were determined by flow cytometric analysis. The peripheral blood samples were collected after 8 h fasting in a tube containing EDTA anticoagulant (BD Vacutainer^®^ EDTA Tubes, BD biosciences, San Jose, CA, USA) and processed within 8 h. Briefly, a layer of blood was added to the same volume of Ficoll Histopaque^®^-1077 (Sigma-Aldrich Inc., St. Louis, MI, USA) in a conical 50 mL tube and centrifuged at 400× *g* for 30–40 min at 20 °C in a swinging-bucket rotor without brake. The mononuclear cell layer was transferred to a new conical 50 mL tube (Corning^®^ 50 mL centrifuge tubes, Sigma-Aldrich Inc.), adding 15 mL RPMI 1640 medium (Gibco-ThermoFisher Inc., Waltham, MA, USA), and centrifuged at 400× *g* for 10 min. For the removal of platelets, we resuspended the cell pellet in 50 mL medium, centrifuged at 200× *g* for 10–15 min at 20 °C and completely removed the supernatant. Live cells were counted in a Burker chamber before and after freezing. Aliquots of 3 × 10^6^ cells were stored in liquid nitrogen after one hour at −20 °C and −80 °C overnight in 2 mL cryopreserved solution containing RPMI 1640 medium (Gibco^®^-ThermoFisher Scientific) 10% FBS and 10% DMSO until the experiments. All experiments were performed after thawing and under the same conditions. The viability of the cells was always greater than 95% after thawing and before the flow cytometric analyses.

### 2.4. Reconstitution of PepTivator^®^ CMV pp65 and IE-1 and Stimulation of PBMCs

To reconstitute the lyophilized peptide pool, the vials were thawed from –20 °C and warmed to room temperature. To dissolve 6 nmol PepTivator^®^ CMV pp65 and 6 nmol PepTivator^®^ CMV IE-1, a sterile syringe (0.5 mL) was filled with 200 μL sterile water, which was injected through the center of the rubber-stopper into the vial containing the lyophilized peptide pool. The solutions were vortexed to completely dissolve the lyophilized peptides with a final concentration of 30 nmol/mL. To avoid repeated freeze–thaw cycles, aliquots of the stock solution were stored at −80 °C.

For stimulation, PBMCs were washed by adding medium and centrifuging at 300× *g* for 10 min. Briefly, the supernatant was aspirated, and the cells were resuspended in culture medium at 10^7^ cells/mL in 96-well plates. Then, 10 μL/mL of each peptide pool stock solution was added to the cell suspensions. The cells were incubated at 37 °C under 5% CO₂ for 6 h and then analyzed by flow cytometry and assayed for secreted cytokines.

### 2.5. Antibodies

The following mAbs were used for the detection of surface markers: anti-CD3 APC-Cy7 (clone: UCHT1), CD14 APC-Cy7 (HCD14), CD16 BV510 (3G8), CD19 APC-Cy7 (HIB19) and CD56 PE-Cy7 (HCD56), all IgG1 isotypes from Biolegend (San Diego, CA, USA). CD57 VioBlue (TB03, IgM isotype) and CD159C PE (REA 205, IgG1 isotype) were purchased from Miltenyi Biotech, Bergisch Gladbach, Germany. CD158a (KIR2DL1) FITC (HP-3E4, IgM isotype) and CD158b (KIR2DL2/DL3) FITC (CH-L, IgG2b isotype) were purchased from BD Biosciences, San Jose, CA, USA.

### 2.6. Flow Cytometric Analysis

#### 2.6.1. NK Cells Gate

The first gate was set on “forward” and “side” scatter, and a second was set to separate CD3+CD19+CD14+ cells (i.e., monocytes, T and B lymphocytes) from CD56+ NK cells. The dot plot was used for NK subsets based on CD56/CD16 expression: CD56^dim^CD16^bright^ (mature phenotype), CD56^bright^CD16^neg/dim^ (immature phenotypes), CD56^neg^CD16^bright^ and CD56^dim^CD16^neg/dim^ (intermediate differentiation).

CD56^dim^CD16^bright^ (mature phenotype) NK cells can be divided into three subpopulations: maturing (NKG2A^pos^KIR2DL1/DL2^neg^), double positive (NKG2A^pos^ KIR2DL1/DL2^pos^) and mature (NKG2A^neg^KIR2DL1/DL2^pos^). In the latter subpopulation, the most mature KIR2DL1/DL2^pos^CD16^bright^ NK cells also express CD57^pos^ and are considered to be the terminally differentiated compartment, whereas the expression of NKG2C distinguishes memory-like cells [21].

#### 2.6.2. T Cells Gate

We studied CD4^+^ and CD8^+^ T cell maturation. A first gate was set on the parameters “forward” and “side” scatter, and a second was set to separate CD3+ cells. Among CD3, we identified CD4^+^ or CD8^+^ lymphocytes. In the CD4+ population, we selected CD45RA^−^ cells that phenotyped as T central memory (Tcm) (CD4^+^CD62L^++^CD27^++^) and T effector cells (CD4^+^CD62L^−^CD27^+/−^). In the CD8+ population, we selected CD45RA^−^ cells that phenotyped as T central memory (Tcm) (CD8^+^ CD62L^++^CD27^++^) and T effector cells (CD8^+^ CD62L^−^CD27^+/−^). Moreover, CD4+ and CD8+ T cells clustered as T naive (Tn) and T stem cell memory (T_SCM_) based on the expression of CD45RA as CD4^+^CD45RA^+^CD27^+++^CD62L^+++^(CD4Tn/scm) and CD8^+^CD45RA^+^CD27^+++^CD62L^+++^ (CD8Tn/scm), respectively [16].

#### 2.6.3. Cell Proliferation Dyes

PBMC were stained with CellTrace™ Violet (CellTrace™ Violet Cell Proliferation Kit, Molecular Probes/Invitrogen) at a final concentration of 2 μM in 96-well cell culture plates according to the manufacturer’s instructions. Briefly, two-well plates were used to detect proliferation: the first was stimulated for 6 h, and the second was unstimulated. After 6 h of stimulation, cells were stained for flow cytometric analysis of proliferation and for phenotype.

### 2.7. Immunoassays

Serum concentrations of biomarkers including IL-6, sFAS, sFASL, granzyme A, granzyme B, perforin and granulysin were quantified by bead-based multiplex LEGENDplex™ analysis (LEGENDplex™ Custom Human Assay, Biolegend) according to the manufacturer’s instructions.

Serum samples taken from aliquots obtained after incubation for QF-CMV assay were used. A mix of 22 CMV peptides and a negative control (no antigens) were thawed at the moment of analysis and used for the detection of biomarkers.

Reactions were run in duplicate with a BD FACSCantoII flow cytometer (BD-Biosciences, San Jose, CA, USA). The data were processed with Legendplex V8.0 software (Biolegend), and concentrations were expressed in pg/mL.

### 2.8. Statistical Analysis

The results were expressed as means and standard deviations (M ± SD) or medians and quartiles (25th and 75th percentiles) for continuous variables as appropriate. A one-way ANOVA non-parametric test (Kruskal–Wallis test) and Dunn test were performed for multiple comparisons. The Wilcoxon signed-rank test and non-parametric tests were used for the response to CMV antigens in a matched pairs set of data. The Kolmogorov–Smirnov test and Shapiro–Wilk test were applied to identify the normal distribution of the data. The Chi-squared test was used for categorical variables. Statistical analysis was performed by SPSS Software (SPSS Inc., Chicago, IL, USA) and graphic representation of the data was performed by GraphPad Prism 9.0 software (Graphpad Holdings, LLC, San Diego, CA, USA). Unsupervised principal component analysis (PCA) was employed to reduce the dimensionality of data hyperspace and for clusterization of the samples based on their cellular composition. The data matrix with variance was constructed with Microsoft Excel and PCA using BioVinci software (BioTuring Inc., San Diego, CA, USA) and ClustVis (http://biit.cs.ut.ee/clustvis/, accessed on 28 March 2021) software. The cellular subsets of patients were also employed to create a decision tree model for the detection of best clustering variables through the Gini criterion. A *p* value of less than 0.05 was considered statistically significant.

## 3. Results

### 3.1. Study Population

A total of 32 participants, 17 awaiting lung transplant and 15 HC, were enrolled in the study. Demographic data, indications for transplant, lung function tests and laboratory findings of the patients are reported in Table 1. There was no significant difference in age, gender prevalence or smoking status between patients and controls (*p* > 0.05 for all). Eight patients (47%) were QF− (IFN-γ < 0.2 IU/mL) before transplant and nine were QF+ (IFN-γ > 0.2 IU/mL). No patients had QF +/− results. In order to obtain the frequency of subjects with humoral/cellular discordance, we determined the agreement between serology and CMV cellular immunity. All patients were on steroid therapy at the time of sampling; seven patients with IPF were on antifibrotics (Nintedanib).

### 3.2. CD4 Teff and Double-Positive NK Cells as the Best Discriminatory Variables between HC and Pre-LTX and QF+ vs. QF− Patients

Cell subsets that emerged from the gate strategy were used to perform PCA analysis in order to highlight populations that distinguished controls from patients and QF CMV-reactive from non-reactive patients.

**Figure 2:**
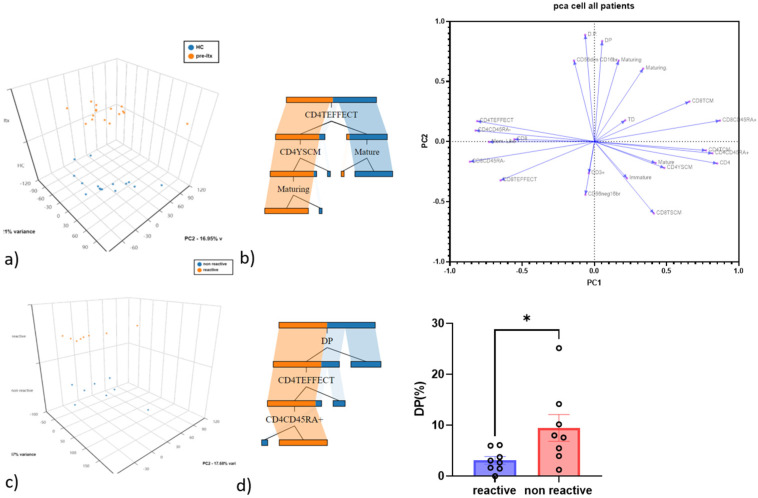
(**a**) Principal component analysis (PCA) of NK cells and T cell subsets. (**b**) The cell subsets of patients were used to build a decision tree model to find the best clustering variables for the two groups. (**c**) Principal component analysis of NK cells and T cell subsets in reactive (QF+) and non-reactive patients (QF−). (**d**) The cell subsets of patients were used to build a decision tree model to find the best clustering variables for the two groups. * *p* < 0.05.

Concerning the PCA between controls and patients, the first and second principal components explained 33.21% and 16.95% of the total variance, CD4 Teff being the best discriminatory variable, as confirmed by decision tree analysis, which was used to determine which variables clustered best by the Gini criterion (Figure 2a,b). The PCA was also used to see the different distributions of T cell and NK cell subsets among patients.

Regarding PCA between QF CMV-reactive and non-reactive patients, the first and second principal components explained 34.37% and 17.68% of the variance, respectively. The best discriminatory variable was double-positive NK cells, as confirmed by decision tree analysis (Figure 2c,d).

### 3.3. Comparison of NK and T Cell Counts in HC and Patients

**Figure 3:**
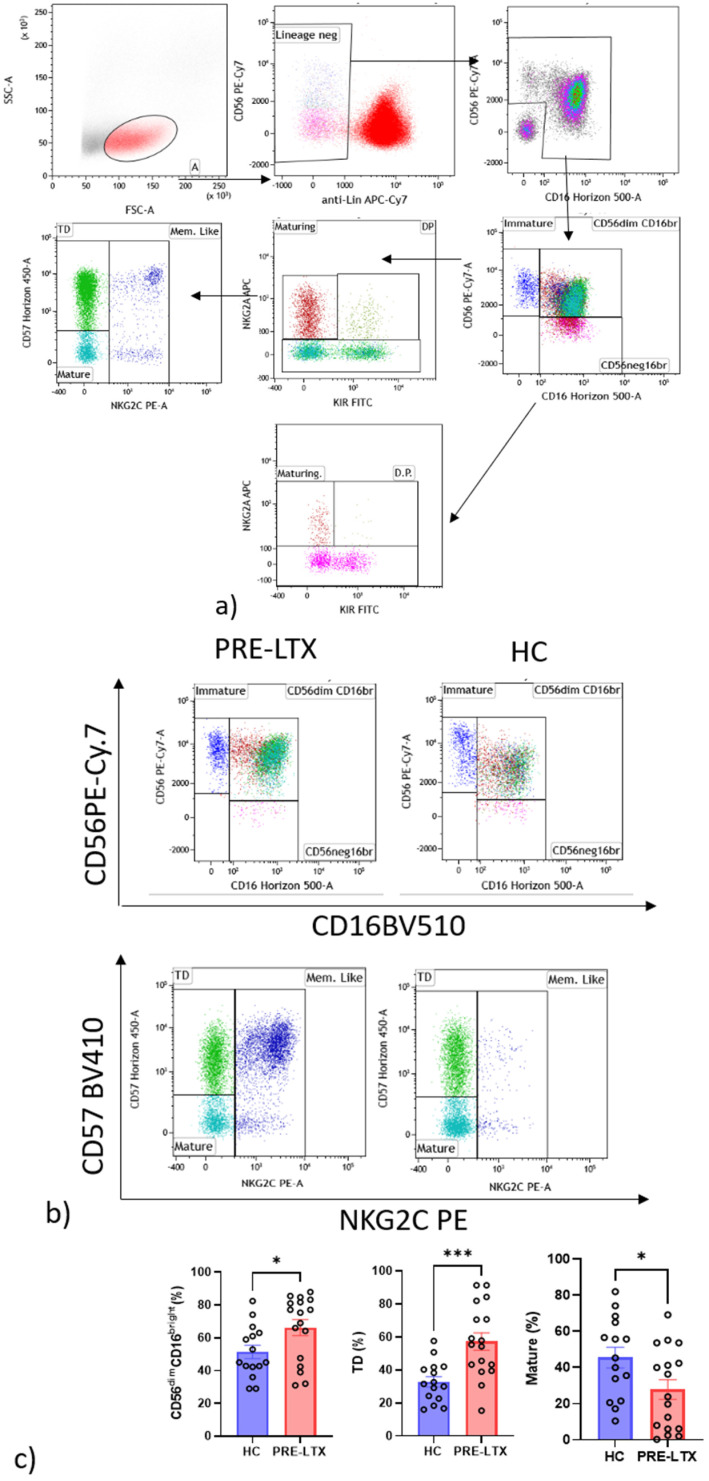
Alteration in the proportion of NK cell subsets expressed in peripheral blood NK cells from pre-LTX (*n* = 17) patients and healthy controls (*n* = 15). (**a**) Gating strategy of NK cells. Frequency of CD56^bright^CD16^low^ immature NK, CD56^dim^CD16^bright^ mature NK and CD56^neg^CD16^bright^ NK cells, and of mature, TD and memory-like NK cells in the two groups. (**b**) Comparison of CD56, CD16, NKG2C and CD57 in HC and pre-LTX. (**c**) Histogram of NK cell expression in the two groups. The level of significance is indicated as follows: * *p* < 0.05 and *** *p* < 0.001.

A comparative analysis was performed to investigate the innate and adaptive immune systems through NK and T cell subsets and functions. We observed that CD56^dim^CD16^bright^ and terminally differentiated (TD) were significantly more numerous in patients than controls, while on the contrary, mature NK were significantly fewer in patients than controls. After dividing the population into QF− and QF+, double-positive NK cells showed a significant increase in QF− patients than in QF+ patients. The other cell subsets did not show any significant differences, as shown in Figure 3a–c.

**Figure 4:**
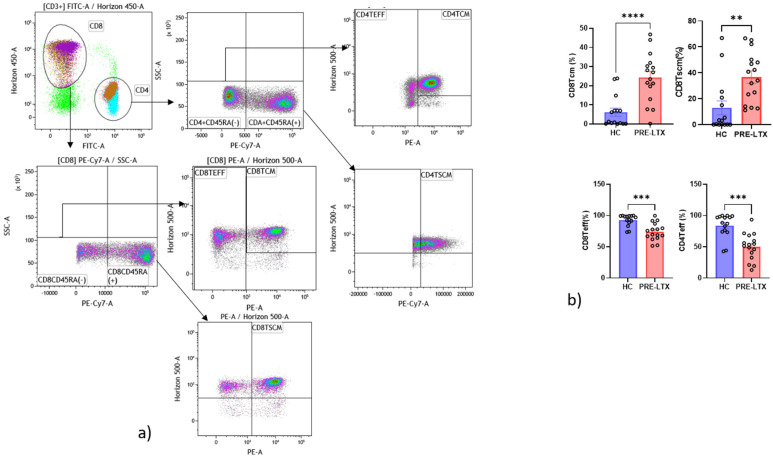
Alteration in the proportion of T cell subsets expressing in peripheral blood from pre-LTX patients (*n* = 17) and HC (*n* = 15). (**a**) Gating strategy of T cells. (**b**) Histogram of comparisons of cell expression in T cells among groups. The level of significance is indicated as follows: ** *p* < 0.01, and *** *p* < 0.001, **** *p* < 0.0001.

Regarding T cell analysis, CD8 Tcm, CD8 Tscm and CD4 Tcm were significantly more numerous in patients than controls, while on the contrary, CD4 Teff and CD8 Teff were significantly fewer. All results are reported in Figure 4a,b.

We also analyzed the subgroups of IPF patients (*n* = 7) and compared them with HC and other patients. No statistical differences emerged between the IPF group and the other patients group as well as with the group of HC.

### 3.4. Stimulation with CMV Antigens, pp65 and IE-1

**Figure 5:**
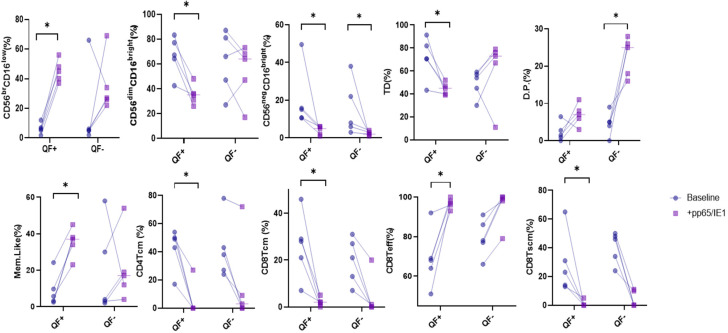
Changes in the proportions of T and NK cell subsets expressed in peripheral blood from pre-LTX patients (*n* = 10) before and after stimulation with pp65 and IE-1. The patients were stratified as QF+ (QuantiFERON-assay positive) and QF− (QuantiFERON-assay negative). * *p* < 0.05.

Cell changes in NK and T cells were also analyzed in 10 patients (5 QF+ and 5 QF−) before and after stimulation with pp65 and IE-1. Considering all 10 patients, we noted a decrease in percentages of the following NK cell subsets after stimulation: CD56^dim^CD16^Bright^, CD56^neg^CD16^bright^, double-positive cells and D.P. cells, and an increase in percentages of immature CD56^Br^CD16^neg^. For T cells, cell analysis after stimulation showed a fall in percentages of CD4, CD4 Tcm, CD8 Tcm and CD8 Tscm and an increase in CD4 Teff and CD8 Teff (Figure 5). Interestingly, when we analyzed NK cell subsets after 6 h of stimulation, no differences were recorded for any cell phenotype.

Regarding the differences between QF+ and QF− patients, in the former, a statistically significant increase in percentages of immature CD56^Br^CD16^neg^, mem-like NK cells and CD8 Teff was recorded after 6 h of stimulation, and there was a decrease in CD56^dim^CD16^Bright^, CD56^neg^CD16^bright^, TD, CD4 Tcm, CD8 Tcm and CD8 Tscm. In the latter, only CD56^neg^CD16^bright^ showed a decrease in percentages, and D.P. cells showed increased expression after stimulation (Figure 5).

### 3.5. Detection of CD8/NK Cytokine Release in Serum Samples and Cell Culture Supernatant

**Figure 6:**
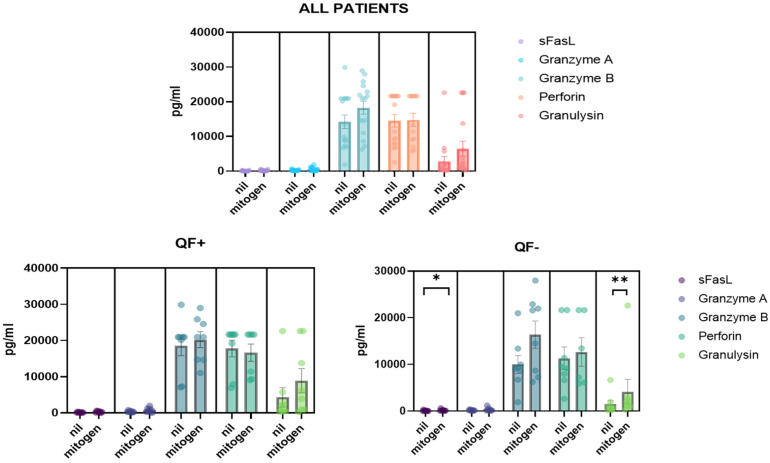
Quantification of cytokines release from CD8 and NK cells in serum of pre-LTX (*n* = 17) patients. Nil: negative control, mitogen: containing a pool of 22 peptides. The level of significance is indicated as follows: * *p* < 0.05, ** *p* < 0.01.

**Figure 7:**
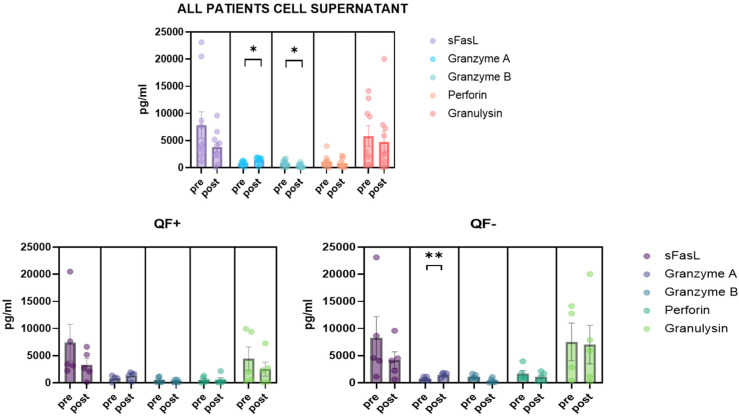
Quantification of cytokines released from CD8 and NK cells in cell supernatant of pre-LTX patients before and after stimulation with CMV antigen. The level of significance is indicated as follows: * *p* < 0.05, ** *p* < 0.01.

Cytokines, including granzyme A/B, granulysin, sFas, sFasL and perforin, were analyzed in the plasma of patients. Quantification was derived from the positive mitogen control (blue cap) and the negative control without antigen (gray cap). Interestingly, the QF− subgroup showed significantly different plasma levels of sFasL and granulysin (Figure 6). Cytokines were also analyzed in cell cultures from the supernatant. In this case, changes in granzyme A and granzyme B were reported before and after stimulation. Dividing the population into QF+ and QF−, the change in granzyme A in the QF− group after stimulation was observed (Figure 7).

### 3.6. Rate of Cell Proliferation before and after Stimulation with pp65 and IE-1

**Figure 8:**
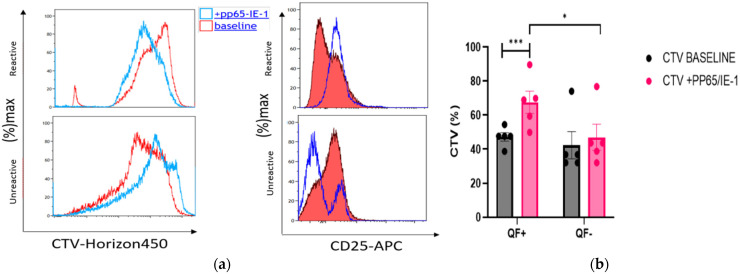

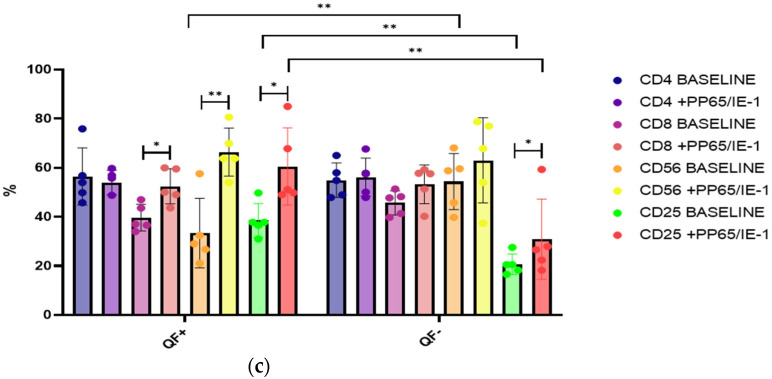
(**a**–**c**) Cell Trace Violet (CTV) was used in QF+ and QF− cells to assay proliferation after stimulation with peptides pp65 and IE-1 for 6 h. Additional markers were used to determine the kinds of cell that proliferated. * *p* < 0.05, ** *p* < 0.01, *** *p* < 0.001.

Cell Trace Violet (CTV) was used to assess the proliferation of cells stimulated with pp65 and IE-1 peptides for 6 h in QF+ and QF− patients. As reported in Figure 8a–c, different rates of proliferation were found between reactive (QF+) and unreactive patients (QF−). Differences emerged also in QF+ and QF− after 6 h of stimulation (Figure 8). In particular, the QF+ group showed an increase in the proliferation rate of CD8, NK and CD25 cells, while the QF+ group only showed increased percentages of CD25+ cells. No differences in CTV percentages were observed at baseline between the two groups. After stimulation, CTV percentages were significantly higher in the QF+ group (Figure 8) than in QF− patients.

## 4. Discussion

Different phenotypes and functions of NK and T cell development were investigated in this study to obtain insights into immunological patterns in patients with end-stage lung disorders, seropositive for CMV and awaiting LTX. We also used QF-CMV to determine which cells were stimulated in the case of CMV infection. Although many studies have focused on CMV and transplant, very few have exploited QuantiFERON CMV, flow cytometric analysis and soluble serum analytes released by activated NK/CD8+ cells to investigate cell subsets that can have a predictive role in the case of CMV infection in patients awaiting LTX. We also investigated the controversial role of QF-CMV assay in CMV-seropositive patients and specific T and NK cell responses to CMV infection when stimulated with peptides pp65 and IE-1. These two peptides were successfully used by Tzannou et al. [22], the pp65 peptide pool being considered the most immunogenic CMV protein [23], and IE-1-specific T cells have been shown to have a protective effect [24]. This evidence prompted us to use the same model.

PCA multivariate analysis showed that cells discriminate controls from patients very well, suggesting different immunological changes in end-stage disease. Notably, CD4 Teff clearly distinguished patients from controls. Teff patrol peripheral tissues and blood and produce effector molecules efficiently when they encounter antigens [25]. Teff cell subsets also support progressive activation [26].

Interestingly, in our cohort of patients, CD4 Teff and CD8 Teff were both fewer. On the contrary, a comparison of CD4 Tcm and CD8 Tcm showed that they were both increased in patients compared with controls. These results are in line with the fact that our cohort was CMV-seropositive. Tcm have high sensitivity to antigen stimulation and provide more effective stimulatory feedback to antigen-presenting cells. After T cell receptor triggering, Tcm mainly produce IL-2, but after proliferation, they efficiently differentiate into effector cells and produce large amounts of IFN-γ or IL-4 [27].

Memory T cells, including Tscm and Tcm, are known for their longevity and high proliferative capacity, which gradually decrease when they differentiate [25]. Oghata et al. [28] showed that at the peak of infection, Teff protect better than long-lived Tcm. However, when we divided our population according to reactivity and non-reactivity to QF-CMV assay, no differences in T cell subsets were found, whereas a specific double-positive phenotype of NK cells differed in the two groups, and it was also the population that best discriminated variables, as illustrated by the decision tree model. These data suggest that at the time of sampling, no patient had active infection.

Let us also highlight a possible bias of our study due to QF-CMV. This in vitro assay measures CMV cell-mediated immunity by quantifying INFγ released by cells after stimulation with a pool of HLA-restricted CMV peptides [29]. Many limits of this method have been reported. In our study, a significant difference between the levels of granulysin and SfasL in the serum of QF− patients (INF-γ levels < 0.2 U/mL) only emerged in mitogen than in nil (negative control).

Regarding the cytokine results, in line with our findings, the literature indicates that granulysin is also a useful biomarker in transplant recipients and is correlated with the severity of graft vs. host disease [30]. In particular, it is considered a marker of acute rejection and steroid resistance [31]. Our QF− subgroup showed an increase in levels of sFasL and granzyme A in cell culture supernatant. Granzyme A is known as a pro-inflammatory mediator in cytokine activity [32]. Granulysin was elevated in QF− patients who are more susceptible to CMV infection. CMV has been associated with acute rejection, and granulysin can be considered a marker of acute rejection. This is a possible link between the risk of rejection and increased susceptibility to CMV.

The study by Zhang et al. raises the possibility that sFasL may contribute to host defenses by aiding the resolution of inflammatory exudate, independently of any effect on viral replication [33]. The lack of CMV-specific cell-mediated immune response detected by QuantiFERON-CMV assay in CMV-seropositive individuals was already reported by Valle-Arroyo et al. [29] CMV infection can change the expression of NK cell membrane receptors, promoting the progressive expansion and persistence of mature phenotypes characterized by CD56^dim^, KIR expression and NKG2A^neg^/CD57^pos^. Our results are intriguing, because double-positive cells are NK cell subsets characterized by the double expression of NKG2A and KIR. This population is considered a maturing “phenotype” and is expressed by mature CD56^dim^CD16^bright^ NK cells. Since KIRs play an established role in the education of NK cells, it was crucial to test their relative functional effects [34]. Beziat’s findings sustain the idea that the dynamics of the NK maturation process may be linked to KIR acquisition and simultaneous NKG2A loss, and it gives rise to NK cells that respond to HLA-E^+^ target cells. The role of NKG2C^pos^ NK cells is associated with the control of CMV infection [35]. NKG2C^pos^CD57^pos^ have several features in common with adaptive immune cells. This explains the name “memory-like or adaptive” NK cell subset [36,37,38,39]. In line with the findings of Bayard et al., the same behavior of NKG2CposCD57pos memory-like NK cells had been observed in our cohort after stimulation with CMV peptides pp65 and IE-1 only in patients considered as “reactive” (QF+) to CMV [40]. Our results confirm that CD57 and NKG2C represent hallmarks of NK cell maturation during CMV infection [41]; however, this is the first time in our knowledge that it was demonstrated in end-stage lung patients who are candidates for LTX.

A paper by Sottile and colleagues demonstrated that similar to NK cells, NKG2C+ is expressed by CD8+ T cells, is associated with HCMV exposure, resides at the boundary between innate and adaptive immunity and exhibited strong effector function against HCMV-infected fibroblasts [42].

After stimulation, we observed a restoration of NK and T cells in QF+ patients, similar to that of the control group, with an increase in Teff and memory-like cells and a decrease in Tcm CD56dimCD16br and TD. In line with the study by Valle-Arroyo et al. [29], the significance of a negative QF result in CMV-seropositive patients needs to be clarified. Interestingly, as found by us, these authors observed that QF− patients showed lower CMV-specific humoral and cellular immune responses than QF+ patients.

Our functional assay also underlined the importance of the proliferative capacity of these cells in response to CMV peptides pp65 and IE-1. Only the QF+ subgroup showed cells with good proliferative properties: in particular, NK, CD8 and CD25 cells proved to be highly proliferative after stimulation.

The limits of our study include the small sample size and monocentric nature of the study, as well as the lack of prior research on specific aspects; however, our patients were well characterized, and the inclusion criteria were well defined. The merits of our research include the enrollment of a prospective cohort of consecutive patients and appropriate analysis of innate and adaptive immunity. Different steps in the development of NK and T cells were also analyzed for insights into what they do in the case of CMV infection.

## 5. Conclusions

This study provides new insights into immunological response to CMV in patients awaiting lung transplant. It confirms that QuantiFERON CMV is more useful than serology for detecting patients who can respond to viral infection. It also provides new evidence of immune dysregulation in patients with end-stage lung disorders, particularly in relation to NK and T cell biology. In particular, reactive patients showed good cell proliferative capacity, especially of CD8 and NK cells.

## Figures and Tables

**Table 1:** Demographic data, Tx indication, therapy and laboratory findings values of selected cohort.

	Pre-LTX (*n* = 17)	HC (*n* = 15)	*p* Values
Age (m ± SD)	58 ± 7.3	52 ± 12	ns
Gender (M:F)	10:7	8:7	ns
Smoking Habits (never/current/former)	5/0/12	5/2/7	ns
Tx indication:			
- IPF	7		
- Emphysema	5		
- DIP	1		
- FIBROSIS + PH	1		
- HP	2		
- PLCH	1		
Therapy (steroids: antifibrotic)	17:7		
Laboratory Findings			
- CRP	1.4 ± 3.7		
- Neutrophils (%)	66.3 ± 15.4		
- Lymphocytes (%)	22.9 ± 12.4		
- Monocytes (%)	7.9 ± 2.7		
- Eosinophils (%)	0.8 ± 0.7		
- Basophils (%)	1.7 ± 1.6		

## Data Availability

The datasets generated during and/or analyzed during the current study are available from the corresponding author on reasonable request.

## Abbreviations

Lung transplant (LTx), solid organ transplant (SOT), cytomegalovirus (CMV), T central memory (Tcm), T effector cells (Teff), peripheral blood mononuclear cells (PBMCs), forced vital capacity (FVC), forced expiratory volume in the first second (FEV1), diffusing capacity of the lung for carbon monoxide (DLCO), Immediate early 1 protein (IE-1).
